# Physiological Responses of Asparagus Plants to Soil Disinfection Strategies Targeting Asparagus Decline Syndrome

**DOI:** 10.3390/plants14131992

**Published:** 2025-06-30

**Authors:** Francisco Javier López-Moreno, Eloy Navarro-León, Miguel de Cara, Teresa Soriano, Juan Manuel Ruiz

**Affiliations:** 1 IFAPA, Institute of Research and Training in Agriculture and Fisheries, 18004 Granada, Spain; franciscoj.lopez.moreno@juntadeandalucia.es (F.J.L.-M.); mteresa.soriano@juntadeandalucia.es (T.S.); 2 Department of Plant Physiology, Faculty of Sciences, University of Granada, 18071 Granada, Spain; jmrs@ugr.es; 3 IFAPA-La Mojonera, Camino San Nicolás n.1, 04745 La Mojonera, Spain; franciscom.cara@juntadeandalucia.es

**Keywords:** *Asparagus officinalis*, antioxidant capacity, biofumigation, oxidative metabolism, phenolic compounds, phytohormones

## Abstract

Asparagus decline syndrome (ADS) poses a significant threat to asparagus cultivation worldwide. To address this challenge, a two-year investigation was carried out in Spain to assess the impacts of three soil disinfection strategies on asparagus crops. These included biofumigation with *Brassica carinata* seed pellets, biofumigation using poultry manure pellets, and chemical disinfection with dazomet. In addition to evaluating the potential of these treatments to alleviate ADS, the research also focused on identifying the physiological changes linked to the syndrome by examining indicators of oxidative metabolism, hormonal equilibrium, and phenolic compound profiles. Among the treatments evaluated, biofumigation with *B. carinata* pellets enhanced vegetative growth, photosynthetic pigment accumulation, antioxidant capacity, and hormonal homeostasis, with these improvements becoming more pronounced in the second year. This approach appeared to promote a healthier physiological status in asparagus plants, likely through improved soil health and reduced biotic and abiotic stress perception. In contrast, chemical disinfection with dazomet, despite initially stimulating some physiological responses, was associated with elevated oxidative stress. Overall, the findings suggest that organic-based soil treatments, particularly *B. carinata* biofumigation, represent a promising strategy to strengthen asparagus vigor and resilience against ADS. Further studies are needed to assess their long-term effects in perennial cultivation systems.

## 1. Introduction

Asparagus (*Asparagus officinalis* L.) is a valuable perennial crop cultivated in many regions around the world. However, its long-term productivity is increasingly compromised by asparagus decline syndrome (ADS), a condition that has become a major limitation to successful cultivation. This syndrome is associated with notable reductions in yield and spear quality [1,2]. Typical symptoms include a general decline in plant vigor, early senescence, and decreased spear emergence, all of which jeopardize commercial asparagus production.

The causes of ADS are diverse and involve a combination of biotic and abiotic stresses. Among the most significant biological agents are pathogenic fungi from the *Fusarium* genus, particularly *F. oxysporum* f. sp. asparagi, *F. redolens*, and *F. proliferatum*, which have been recognized as major contributors to the syndrome [3,4]. These organisms infect the vascular tissues of asparagus, disrupting the transport of water and nutrients, and giving rise to symptoms such as root and crown rot, wilting, and plant death [5]. Moreover, additional soilborne pathogens and parasitic nematodes can further aggravate decline symptoms by damaging root systems and increasing the plant’s vulnerability to infection [6].

Abiotic factors are also crucial factors in the development and severity of ADS. Soil degradation, including nutrient depletion and poor soil structure, can contribute to plant stress and increase vulnerability to disease [7]. The depletion of essential mineral nutrients, such as N, P, and K, can reduce plant vigor and compromise physiological processes critical for growth and disease resistance. Additionally, the accumulation of herbicide residues and alterations in soil structure may further exacerbate stress conditions, reducing asparagus resilience to ADS [8]. Water availability is another critical factor, as drought conditions intensify oxidative stress, leading to the excessive reactive oxygen species (ROS) accumulation that damages cellular components and accelerate plant senescence [9,10]. High temperatures and fluctuating environmental conditions may further compromise plant resilience, influencing hormonal imbalances and metabolic disruptions that predispose asparagus to ADS [1]. Moreover, winter crown injuries and excessive cropping pressure have been suggested as additional contributors to the decline of asparagus fields [11].

Given the importance of ADS, several management strategies have been developed to reduce its impact. Breeding programs have focused on selecting resistant cultivars, but genetic resistance to *Fusarium* spp. remains a challenge due to the pathogen’s high diversity and adaptability [12]. Some cultivars, such as Plasenesp and Dariana, have proved lower susceptibility to *F. solani*, offering potential alternatives for cultivation in ADS-affected areas [13]. However, further research is required to develop more robust resistance mechanisms, particularly against *F. oxysporum* [14].

Chemical control strategies, such as soil fumigation, have been employed to manage *Fusarium* populations, with dazomet being one of the most commonly used fumigants [15]. While effective in reducing pathogen inoculum levels, chemical fumigation has environmental and regulatory limitations, prompting the search for alternative control measures [3]. One promising approach is the use of organic amendments, such as olive-mill compost and poultry manure pellets, which have shown potential in suppressing *Fusarium* spp. through soil microbiome modulation and improved soil health [5,16]. Organic amendments also contribute to soil fertility by improving soil structure, replenishing essential nutrients, and enhancing microbial activity, which can indirectly reduce the impact of ADS [17].

Another alternative strategy is biosolarization, which combines soil solarization with the incorporation of organic amendments to enhance pathogen suppression. The integration of organic amendments, such as poultry manure and olive-mill compost, not only promotes beneficial microbial communities but also improves soil fertility, potentially alleviating the negative effects of ADS [5]. The use of Brassicaceae plants such as *Raphanus*, *Sinapis*, and *Eruca* in biofumigation practices has attracted increasing interest due to their capacity to release isothiocyanates, compounds with recognized antifungal activity, when their tissues undergo enzymatic degradation. Beyond their biocidal action, these species also enrich the soil with organic matter and nutrients, which can enhance soil structure and fertility, ultimately contributing to increased plant tolerance to ADS [17]. Nevertheless, the success of biofumigation is influenced by factors such as soil type, climatic conditions, and the particular *Fusarium* populations involved, making it essential to validate its efficacy under specific local conditions [18].

In response to the ongoing challenges posed by ADS, a two-year field experiment was conducted in Spain to assess the impact of three soil treatment strategies on asparagus plants: poultry manure-based biofumigation, biofumigation using *Brassica* seed pellets, and chemical soil disinfection with dazomet. Alongside evaluating their ability to alleviate ADS symptoms, the study also aimed to characterize the physiological disturbances associated with the syndrome by measuring markers related to oxidative metabolism, hormone regulation, and phenolic content. This approach was designed to improve our understanding of ADS pathophysiology and to inform the development of sustainable management practices for affected crops.

## 2. Results

### 2.1. Biomass Production

**Figure 1:**
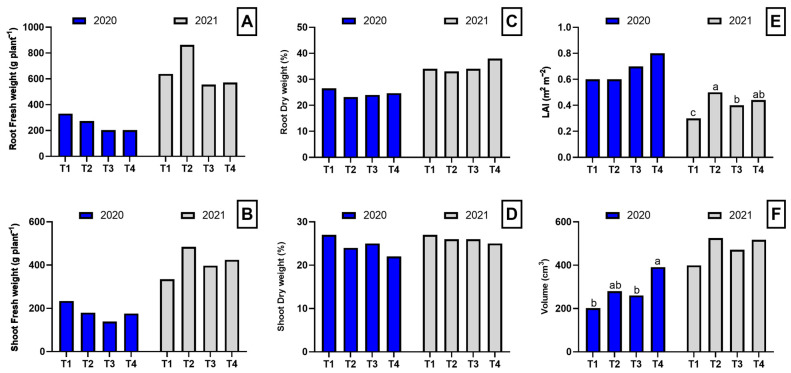
Biomass parameters in the root and shoot of asparagus plants subjected to the different treatments tested during two years of study (2020–2021). Root fresh weight (**A**), shoot fresh weight (**B**), toot dry weight (**C**), shoot dry weight (**D**), leaf area index (**E**), and volume (**F**). Values with different letters indicate significant differences. Column groups without letters indicate no significant differences among treatments. The applied treatments were: control without any application (T1), biosolarization with *Brassica* pellets (T2), biosolarization with poultry manure pellets (T3), and chemical disinfection with dazomet (T4).

In general, the analysis of biomass parameters showed no statistically significant effects on biomass or dry matter content in roots and shoots in either of the evaluated years. In 2020, no significant differences were observed in root fresh weight, shoot fresh weight, root dry weight percentage, or shoot dry weight percentage among treatments. However, plant volume showed significant differences, with dazomet exhibiting the highest value (391 cm^3^), significantly higher than control and biofumigation with poultry manure, while biofumigation with *B. carinata* showed an intermediate value. In 2021, root fresh weight, shoot fresh weight, and dry matter percentages again showed no significant differences among treatments. However, the leaf area index (LAI) presented highly significant differences, with T2 showing the highest value, followed by T3 and T4, while T1 had the lowest value. Plant volume, although not significantly different, tended to be higher in T2 and T4 compared to T1 and T3 (Figure 1).

### 2.2. Photosynthetic Pigments

In the first year, plants from plots treated with *Brassica* pellets exhibited the highest total chlorophyll content, while those from the untreated control, chicken manure pellet, and dazomet-treated plots showed significantly lower and similar values. The chlorophyll a/b ratio was notably higher in plants from plots treated with dazomet, whereas the other treatments resulted in lower and comparable values. Carotenoid content was also highest in plants from *Brassica*-treated plots, followed by those from dazomet-treated plots. Meanwhile, plants from the untreated control showed intermediate carotenoid levels, and those from plots treated with chicken manure pellets had the lowest values (Table 1).

In the second year, plants from *Brassica*-treated plots once again showed the highest total chlorophyll content, whereas those from the untreated control had the lowest values. Plants from plots treated with chicken manure pellets and dazomet exhibited intermediate total chlorophyll levels, significantly lower than those from *Brassica*-treated plots but higher than those from the untreated control. The chlorophyll a/b ratio was significantly lower in plants from the untreated control, while the other treatments resulted in similar and higher values. Regarding carotenoids, plants from *Brassica*-treated plots had the highest content, followed by those from plots treated with dazomet and chicken manure pellets, which showed comparable values. In contrast, the lowest carotenoid concentration was observed in plants from the untreated control (Table 1).

### 2.3. Oxidative Metabolism

**Figure 2:**
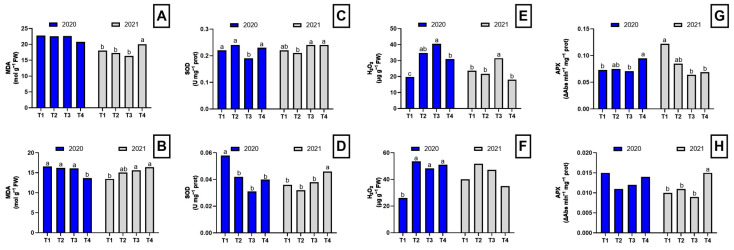
Oxidative stress parameters in the root and shoot of asparagus subjected to the different treatments tested during two years of study (2020–2021). Root MDA concentration (**A**), shoot MDA concentration (**B**), root SOD activity (**C**), shoot SOD activity (**D**), root H_2_O_2_ concentration (**E**), shoot H_2_O_2_ concentration (**F**), root APX activity (**G**), shoot APX activity (**H**). Values with different letters indicate significant differences. Column groups without letters indicate no significant differences among treatments. The applied treatments were: control without any application (T1), biosolarization with *Brassica* pellets (T2), biosolarization with poultry manure pellets (T3), and chemical disinfection with dazomet (T4).

In the first year, the dazomet-treated plants exhibited lower MDA concentrations in the roots compared to the other treatments, although these differences were not statistically significant (Figure 2A). However, in the aerial part of the plant, significant differences were observed among treatments (Figure 2B). In the second year, a different pattern in MDA concentration was observed, with dazomet-treated plants showing significantly higher MDA levels in both roots and shoots (Figure 2).

Regarding SOD activity, in the first year, plants from plots treated with chicken manure pellets showed the lowest root SOD activity. In contrast, in the aerial part of the plant, control plants displayed significantly higher SOD activity compared to the other treatments. In the second year, plants from plots treated with *Brassica* pellets exhibited lower SOD activity in the roots. However, in the aerial part, SOD activity was higher in dazomet-treated plants compared to the other treatments (Figure 2).

Significant differences in H_2_O_2_ concentration were detected in the first year in both roots and aerial parts of asparagus plants. In the roots, plants from plots treated with *Brassica* and chicken manure pellets showed higher H_2_O_2_ concentrations than the control and dazomet-treated plants (Figure 2E). In the aerial part, all three disinfection treatments resulted in an increase in H_2_O_2_ levels (Figure 2F). In the second year, plants from plots treated with chicken manure pellets showed the highest H_2_O_2_ concentrations in the roots, while no significant differences were found in the aerial part. However, plants from plots treated with pellets continued to show the highest values (Figure 2).

In the first year, no significant differences were found in APX activity in the aerial part, but higher APX activity was observed in the roots of plants from plots treated with dazomet and *Brassica* pellets. In the second year, plants from control and *Brassica* pellet-treated plots exhibited the highest APX activity in the roots (Figure 2G). In contrast, in the aerial part, plants from plots treated with chicken manure pellets and dazomet showed the highest APX activities (Figure 2H).

**Figure 3:**
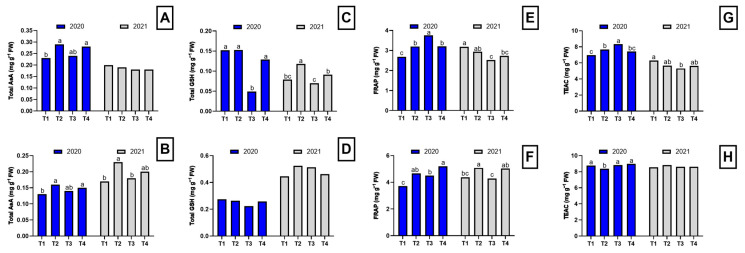
Antioxidant compound concentration and antioxidant test in the root and shoot of asparagus subjected to the different treatments tested during two years of study (2020–2021). Root total AsA concentration (**A**), shoot total AsA concentration (**B**), root total GSH concentration (**C**), shoot total GSH concentration (**D**), FRAP test in the root (**E**), FRAP test in the shoot (**F**), TEAC test in the root (**G**), TEAC test in the shoot (**H**). Values with different letters indicate significant differences. Column groups without letters indicate no significant differences among treatments. The applied treatments were: control without any application (T1), biosolarization with *Brassica* pellets (T2), biosolarization with poultry manure pellets (T3), and chemical disinfection with dazomet (T4).

In the first year of the study, plants from plots treated with *Brassica* pellets, chicken manure pellets, and dazomet showed higher total ascorbate (AsA) levels in both roots and shoots compared to the control plants (Figure 3). This suggests that the disinfection techniques used may promote a greater accumulation of AsA in asparagus plants. Ascorbate, also known as vitamin C, is an important antioxidant that plays a crucial role in protecting plants against oxidative stress.

Regarding GSH levels, in the first year, similar concentrations were observed in the roots of control plants, *Brassica* pellet-treated plants, and dazomet-treated plants, while plants from plots treated with chicken manure pellets exhibited significantly lower levels (Figure 3C). In the aerial part, no significant differences in GSH levels were found among treatments, although the accumulation pattern remained the same (Figure 3D). These results indicate that chicken manure pellet treatment may negatively affect GSH levels in asparagus roots. However, in shoots, no significant differences were observed among treatments, suggesting that the antioxidant response may vary between plant organs.

In the second year of the study, the GSH accumulation pattern in roots was similar to that of the first year, but significant differences were observed among all treatments (Figure 3C). Plants from plots treated with *Brassica* pellets exhibited the highest total GSH levels in roots compared to the other treatments. In contrast, no significant differences in GSH levels were found among treatments in the aerial part during the second year (Figure 3D).

In the first year, plants from plots treated with chicken manure pellets showed the highest Ferric Reducing Ability of Plasma (FRAP) values in roots, whereas in shoots, the highest values were observed in plants from plots treated with *Brassica* pellets and dazomet (Figure 3). These results suggest that these treatments may have stimulated greater antioxidant capacity in plant tissues. In the second year, control plants and *Brassica* pellet-treated plants exhibited the highest FRAP values in roots, while in the aerial part, the highest values were found in plants from plots treated with *Brassica* pellets and dazomet (Figure 3).

Regarding Trolox Equivalent Antioxidant Capacity (TEAC), its trend followed that of FRAP in terms of the treatments that exhibited the highest values in the two study years for roots. In shoots, a similar trend was observed across both cycles, with dazomet-treated plants showing the highest values in the first year and *Brassica* pellet-treated plants in the second year (Figure 3).

### 2.4. Phytohormone Profile

The results for phytohormone concentrations are described in Table 2. In 2020, plants grown in plots amended with *Brassica* pellets exhibited reduced jasmonic acid (JA) concentrations in their roots compared to those under the other treatments. This trend was less pronounced in the following year, with no statistically significant differences detected, although slightly lower JA levels persisted in plants treated with *Brassica* pellets and dazomet.

As for abscisic acid (ABA) levels in roots, a decline was observed in plants receiving the *Brassica* pellet treatment, whereas an increase was recorded in those exposed to dazomet, relative to both the control and the poultry manure pellet treatments.

In the shoot, most phytohormones showed no significant variation among treatments across both years. Nevertheless, in 2020, higher GA_4_ levels were detected in plants from the poultry manure pellet treatment. In contrast, reduced indoleacetic acid (IAA) levels were found in plants subjected to both *Brassica* and poultry manure pellet applications.

By 2021, notable differences in shoot hormone levels emerged for jasmonic acid and salicylic acid (SA). Specifically, JA levels decreased and SA levels increased in plants treated with *Brassica* pellets when compared with the untreated control.

### 2.5. Phenolic Compounds Profile

In the root, only two of the evaluated compounds were detected. In 2020, plants from *Brassica* pellet-treated plots showed the lowest levels of caffeic acid. Meanwhile, plants from dazomet-treated and control plots exhibited the highest levels of this compound. In 2021, similar patterns were observed for caffeic acid, but without significant differences, while plants from plots treated with dazomet had the lowest levels of caffeic acid hexose (Table 3).

In the shoot, a greater number of compounds were detected. In 2020, plants from chicken manure pellets showed the higher feruloil-QA concentration compared to the other treatments. Additionally, plants from dazomet-treated plots exhibited the highest levels of p-coumaric acid derivative and ferulic acid derivative, while no significant differences were found for 4-CQA (Table 3).

Regarding root flavonols, they were below the detection level in both 2020 and 2021. However, in the shoot, several flavonols were detected. Plants from *Brassica* pellet-treated plots exhibited the lowest levels of Q-rutin, and kaempferol rutinoside but higher Q-Rutin-hexose in comparison to plants from the control plot. Plants from both the *Brassica* and chicken manure pellets treatments showed lower Quercetin rutinoside derivative. Meanwhile, plants from dazomet-treated plots had the highest levels of Q-rutin and Q-rutin-hexose. Additionally, plants from chicken manure pellet- and dazomet-treated plots showed the highest levels of isorhamnetin rutinoside (Table 3). In 2021, plants from control plots showed the highest levels of vicenin 2. In addition, plant from *Brassica* pellets and dazomet plots showed lower levels of Q-rutin (Table 3).

### 2.6. Mineral Nutrient Profile

In 2020, all treatments increased root K levels compared to the control plants. The application of *Brassica* pellets and chemical disinfection with dazomet led to an increase in Ca, Mg, and Fe concentrations in asparagus roots, whereas the application of chicken manure pellets resulted in a reduction of N and *p* levels compared to the control plots. In 2021, *Brassica* pellet application increased root Ca concentrations, chicken manure application enhanced Mg and N levels, and dazomet disinfection raised Ca, Mg, and Mn concentrations relative to the control (Table 4).

Regarding the shoots, in 2020, dazomet application reduced the accumulation of N, P, K, and Zn, while chicken manure application also decreased N levels compared to plants from the control plots. In 2021, all treatments increased Mg concentrations in the shoots relative to the control. The application of *Brassica* pellets led to a reduction in S, Ca, Mn, Cu, and B levels. Chicken manure application also reduced Zn concentrations, and chemical disinfection with dazomet decreased the accumulation of N, P, S, Mn, Zn, Cu, and B (Table 4).

## 3. Discussion

The results showed that the evaluated treatments did not have a pronounced effect on asparagus growth parameters during these first two years. Nevertheless, an increase in vegetative density, particularly under *B. carinata* pellet treatment in the second year, suggests that this strategy may have had a positive influence on early plant development. In perennial crops like asparagus, where initial vegetative vigor is a key determinant of future spear yield [19], such physiological responses may serve as early indicators of improved productivity in later harvest cycles. These findings are consistent with previous studies showing that organic amendments and biofumigation can enhance vegetative growth and reduce soil-borne pathogen pressure [17,20,21]. It is also possible that improvements in physiological indicators in plants treated with *B. carinata* reflect a priming effect that prepares the plant for enhanced performance over time, even if such advantages have not yet translated into measurable biomass increases. This highlights the importance of evaluating asparagus response across multiple growing seasons, particularly given the crop’s long production cycle and delayed yield stabilization. Moreover, biomass measurements alone may not fully capture changes in root architecture, nutrient uptake efficiency, or carbon allocation patterns, all of which can contribute to long-term vigor and productivity. Future studies should incorporate more comprehensive growth and yield metrics over successive years to better assess the agronomic value of these treatments.

Regarding photosynthetic pigments, a clear positive effect of *B. carinata* pellets was observed, with all three treatments showing beneficial effects in the second year. The incorporation of *B. carinata* into the soil may enhance pigment synthesis and accumulation in asparagus plants. This effect could be related to the presence of bioactive compounds, such as glucosinolates and other secondary metabolites [22,23], and also the mineral nutrients [24], which might influence pigment metabolism and improve the photosynthetic response. Chlorophylls play a key role in light capture and energy transformation, and their accumulation is typically linked to improved plant performance and stress tolerance [25]. The higher pigment concentrations found in *B. carinata*-treated plants could reflect enhanced chloroplast development or a preservation of pigment biosynthesis pathways under reduced stress conditions, potentially mediated by improvements in soil health or microbial dynamics, as reported in other crop systems [26,27]. Furthermore, the elevated carotenoid content in this treatment may indicate stronger antioxidant defenses, contributing to greater tolerance to biotic stress factors such as those associated with ADS [10]. The intermediate pigment values observed in plants treated with dazomet and chicken manure pellets also suggest a moderate physiological benefit, although less pronounced than with *B. carinata*.

Similarly, the chemical disinfection treatment with dazomet also exhibited high Chl a/b values in the first year. This may indicate that dazomet, despite being a chemical compound, had a stimulating effect on the accumulation of Chl a relative to Chl b. All disinfection treatments increased the Chl a/b ratio, thereby enhancing the accumulation of Chl a, the primary pigment of photosystems I and II. A higher Chl a/b ratio is often associated with a better stress adaptation, as Chl a is mainly located in the reaction centers, facilitating charge separation and electron transport, while Chl b is largely bound to the light-harvesting antenna complexes. Therefore, an increased Chl a/b ratio suggests a shift towards a greater proportion of active reaction centers relative to antenna pigments, potentially reflecting enhanced photosynthetic efficiency and improved resilience to stress conditions [28].

Oxidative stress showed notable fluctuations between growing seasons and between root and shoot tissues, reflecting dynamic plant responses to fluctuating stressors. The elevated FRAP and TEAC values detected following biofumigation treatments suggest that these strategies enhance the overall antioxidant potential of asparagus plants, likely by stimulating both enzymatic and non-enzymatic defense mechanisms [29,30]. The initial rise in H_2_O_2_ under biofumigation treatments (with both *B. carinata* and chicken manure pellets) likely served as a signaling cue, triggering enhanced synthesis of antioxidant defenses, including AsA, a primary mediator of H_2_O_2_ detoxification [30]. The concurrent rise in antioxidant molecules may indicate an induced redox homeostasis aimed at maintaining ROS below damaging thresholds while enabling their signaling functions. The subsequent decline in both H_2_O_2_ and AsA during the second year suggests acclimation, where plants achieved a more efficient ROS-scavenging equilibrium over time. In addition, the enhanced supply of mineral nutrients and organic matter provided by this treatment may contribute to reducing oxidative stress in plants, as reported in previous studies [31].

Conversely, dazomet treatment continually elevated oxidative markers across seasons, implying a sustained imbalance in redox homeostasis. This chronic oxidative burden likely stems from disruptions to beneficial soil microbial populations, which can undermine the plant’s resilience mechanisms. This observation aligns with existing literature positing that soil disinfection chemicals can stimulate the production of ROS, culminating in lipid peroxidation and oxidative damage [21,32].

Regarding GSH levels, biofumigation with chicken manure pellets resulted in significantly lower GSH concentrations in the roots compared to the other treatments. This result suggests that this treatment may negatively impact the root antioxidant system by reducing GSH availability. In contrast, in the second year, biofumigation with *B. carinata* pellets promoted the highest total GSH levels in roots, suggesting strengthened redox buffering, improved detoxification, and enhanced stress resilience. This increase in GSH may reflect an enhanced ability of plants under this treatment to respond to oxidative challenges and recover from stress conditions, given the importance of this antioxidant [33].

These responses showcase how initial ROS peaks could act as priming signals, activating antioxidant enzymes, refining ROS management, and reinforcing defense pathways. Over time, this priming may lead to improved physiological homeostasis and productivity under biotic pressure like ADS. By fortifying redox balance, especially via GSH and AsA cycles, plants likely bolster their resistance to subsequent stressors, promoting sustainable yield stability. In summary, the cultivar of antioxidant capacity via biofumigation with *B. carinata* could be a proactive stress management strategy, offering long-term resilience benefits. This contrasts with chemical disinfection, which may provoke chronic oxidative stress, potentially compromising plant health and yield sustainability over time.

Considering phytohormone profile, in 2020, the application of the different soil treatments did not cause major alterations in the hormonal profile of the asparagus plants. Nonetheless, slight changes, such as the higher GA_4_ levels in plants treated with chicken manure pellets and the reduction of IAA under biofumigation with *B. carinata* pellets, point to possible early effects on growth regulation and root development, respectively [34]. By contrast, in 2021, clearer treatment-dependent hormonal responses were detected. Thus, the decrease in JA and ABA levels observed in plants from the plots biofumigated with *B. carinata* pellets indicates a lower perception of biotic and abiotic stresses, supporting the hypothesis that biofumigation improved soil health and reduced the need for defense-related hormonal signaling. Conversely, the chemical disinfection with dazomet resulted in an increase in both JA and ABA, suggesting that although pathogen pressure might have been alleviated, this treatment could have led to soil disturbances that triggered abiotic stress responses. This is supported by other studies that related the concentration of these hormones to stress levels [35,36].

The results of phenolic compounds profile showed that the differential accumulation of these compounds in asparagus plants highlights the influence of soil disinfection treatments on secondary metabolism. In general, the aerial parts of the plants accumulated higher levels of phenolic compounds than the roots, consistent with previous studies indicating that phenolic biosynthesis is primarily localized in aboveground tissues [37]. This pattern reinforces the protective role of phenolics in photosynthetic tissues against oxidative stress and pathogen attack [38].

The lower caffeic acid levels in roots observed under *Brassica* pellet treatment suggest a reduced activation of lignin biosynthesis and other phenylpropanoid-related defenses, likely reflecting a diminished perception of biotic stress [39]. Given the known role of caffeic acid in reinforcing cell walls and deterring pathogens [37], this reduction could indicate that plants under *Brassica* biofumigation experienced improved soil conditions or a less hostile microbial environment, allowing a shift in metabolic priorities.

The enhanced accumulation of hydroxycinnamic acid derivatives, such as feruloylquinic acid and p-coumaric acid derivatives, particularly under dazomet and chicken manure treatments, may represent an adaptive strategy to cope with oxidative stress or altered microbial dynamics [40]. These compounds contribute to cell wall fortification and ROS scavenging [41], and their upregulation may reflect compensatory defense responses triggered by chemical disruption of the soil microbiota or by the transient stress associated with organic amendments.

The patterns observed in shoot flavonol content further support the idea that soil disinfection treatments influence secondary metabolism through both stress signaling and resource allocation. The reduced levels of quercetin and kaempferol derivatives in *Brassica*-treated plants could imply a reallocation of metabolic flux away from flavonoid synthesis under improved physiological status. However, the increased accumulation of Q-rutin-hexose in these plants might reflect modifications in glycosylation processes, which can regulate antioxidant activity and compound transport [42]. The presence of glucosinolate breakdown products in *Brassica*-treated soils may also modulate flavonoid biosynthesis, either through direct signaling or by altering microbial-mediated pathways [43].

In dazomet-treated plants, the consistently higher levels of flavonols such as quercetin rutinoside and isorhamnetin rutinoside suggest persistent oxidative pressure, likely stemming from disrupted soil microbial equilibrium or phytotoxic byproducts of the chemical itself. These compounds are involved in ROS neutralization and in enhancing pathogen resistance [44], indicating that plants under dazomet treatment maintained a primed state of defense, potentially at the cost of growth or metabolic efficiency.

The decreased accumulation of vicenin-2 in *Brassica*- and dazomet-treated plants may further support the hypothesis of a shift in defense strategy or the suppression of certain branches of the flavonoid pathway. As a known antioxidant and antimicrobial agent, vicenin-2 is often associated with constitutive defense in aerial tissues, and its downregulation may reflect changes in signaling dynamics or metabolic prioritization under altered soil conditions [45].

Interestingly, the application of soil treatments appeared to negatively affect the accumulation of specific flavonoids such as apigenin derivatives in the shoot. Given that apigenins are known for their antioxidant and defensive roles [46], a reduction in their levels may reflect a shift in the plant’s defense strategy or resource allocation under altered soil conditions.

Overall, the treatments applied in this study influenced nutrient accumulation in asparagus plants, particularly promoting Mg enrichment. This consistent increase in Mg concentrations across treatments and years highlights the potential of soil amendments to enhance the availability or uptake of this essential element, which plays a crucial role in chlorophyll structure and various enzymatic processes [47].

Among the treatments, chicken manure pellets had the least impact on nutrient accumulation, with only minor alterations observed in both root and shoot tissues. This suggests that organic amendments based on chicken manure might maintain a more balanced nutrient profile in asparagus, avoiding excessive shifts in nutrient availability or plant uptake patterns. The stabilizing effect of chicken manure on soil nutrient dynamics has been noted in other cropping systems [48], supporting its use for improving soil fertility without inducing nutrient imbalances. In contrast, the application of *Brassica* pellets exerted a more pronounced effect on root nutrient accumulation. Increases in Ca, Mg, and Fe concentrations in the roots following *Brassica* application indicate that biofumigation not only contributes to pest and pathogen suppression [49] but may also improve root nutrient uptake, possibly through modifications in the soil microbial community or root architecture. In addition, the direct supply of these elements by the treatment and the higher content of organic matter could have improved nutrient uptake. Enhanced nutrient accumulation after *Brassica* amendments has been previously reported in other crops such as tomato and broccoli [50,51], suggesting a broader applicability of this strategy for improving plant mineral nutrition.

Conversely, the chemical disinfection treatment with dazomet had detrimental effects on shoot nutrient concentrations, particularly reducing the accumulation of essential macronutrients (N, P, K, S) and micronutrients (Zn, Mn, Cu, B). The use of dazomet, while effective for pathogen control, may disrupt soil microbial communities [40], leading to impaired nutrient cycling and reduced nutrient availability for the plant. In addition, this treatment did not present an extra supply of mineral nutrients like the other two treatments. Similar negative impacts of soil fumigation on nutrient uptake and plant growth have been documented in crops such as strawberry and lettuce [52].

## 4. Materials and Methods

### 4.1. Plan Material and Experimental Setup

The plant material used in this study was *A. officinalis* L., var. Grande. Seeds were sourced from the seed supplier INTERSEMILLAS S.A. (Valencia, Spain) and sown in a nursery on 7 March 2018, using forestry trays. The seedlings were later transplanted into field plots affected by decline syndrome on 11 June 2018. The planting design consisted of 1.3 m spacing between rows and 0.33 m between individual plants.

In the experimental plot, irrigation was managed through the traditional method of furrow flooding, beginning after the harvest season ended (around May) and continuing until the crop approached senescence (from mid to late August). Water applications were scheduled approximately every 20 days, although adjustments were made based on weather conditions and the soil’s ability to retain moisture. Fertilization practices involved the use of commercial fertilizers supplying macro- and micronutrients, such as ammonium sulfate nitrate (FERTIBERIA S.A., Madrid, Spain) and KNO_3_ (Multi-K^®^, Haifa Iberia, Madrid, Spain). Multi-K^®^ contains 46% potassium oxide and 13% nitrogen in the form of potassium nitrate. Granular fertilizers were uniformly applied in early February, prior to spear emergence. Ammonium sulfate nitrate and potassium nitrate were used as primary sources of N, S, and K. After application, fertilizers were incorporated into the soil through mechanical tillage. Whenever possible, this operation was scheduled just before anticipated rainfall to facilitate nutrient integration into the soil or immediately after rainfall to exploit the existing soil moisture for nutrient dissolution, reducing the need for additional soil disturbance.

The field trial was established in an asparagus-growing region in the province of Granada, southern Spain, specifically in El Jau (37°11′58.4′′ N, 3°44′23.4′′ W). A randomized complete block design was employed, comprising four treatments with four replicates each, resulting in a total of 16 plots. Each plot measured 12 × 4.2 m^2^ and was separated from adjacent plots by a 2 m buffer zone to limit treatment interference. To avoid cross-contamination via irrigation runoff, 50 cm tall and wide soil ridges were constructed around each plot. Each experimental unit contained 108 transplanted asparagus plants.

The treatments under evaluation comprised: (T1) a control without soil disinfection; (T2) biofumigation with *B. carinata* seed pellets (Biofence^®^, Triumph Italia, Cerealtoscana Group, Livorno, Italy) at a rate of 5000 kg/ha, with a nutrient composition of 6% N, 7% P_2_O_5_, 4.4% SO_3_, 2.6% K_2_O, 0.9% MgO, and 84.2% organic matter; (T3) biofumigation with poultry manure pellets (Riger^®^, Ferm O Feed, Helmond, The Netherlands) also at 5000 kg/ha, containing 4% N, 3.4% P_2_O_5_, 3.2% K_2_O, 0.9% MgO, 65% organic matter, and 8% Ca; and (T4) chemical soil disinfection with dazomet (Basamid^®^, Certis Europe, Elche, Spain) at 600 kg/ha, with a formulation consisting of 98% dazomet.

All plots had previously shown severe symptoms of asparagus decline, including high plant mortality. On 4 May 2018, the organic treatments (*Brassica* and poultry manure pellets) were manually and evenly distributed across the respective plots, while dazomet was applied using a calibrated manual spreader. Following treatment application, the soil was rotavated to approximately 20 cm depth to ensure uniform incorporation of the amendments, followed by a light irrigation (~10 mm) to activate the bioactive compounds. A 120-gauge transparent totally impermeable film (TIF) was then placed over the plots and buried at the edges to ensure airtight sealing. The film remained in place for 25 days (4–29 May 2018). During this period, average daily air temperatures ranged between 15 and 18 °C, with maximum values exceeding 24 °C on several days. Relative humidity was generally high, averaging over 70%, and cumulative precipitation was low (59.6 mm), with most days remaining dry. The meteorological information was obtained from the publicly available database managed by the Spanish Ministry of Agriculture, Fisheries, and Food (https://servicio.mapa.gob.es/websiar/SeleccionParametrosMap.aspx?dst=1, accessed on 25 June 2025).

### 4.2. Sampling and Biomass Parameters

Two years after crown transplantation, asparagus plants were sampled by randomly selecting twelve individuals from each plot. Individuals were randomly selected from the central area of each plot to avoid border effects, maintaining a minimum buffer distance of approximately one meter from the plot edges. Sampling was conducted in the early morning to minimize diurnal variation in physiological parameters. On the day of sampling, plant volume was estimated by measuring the average height and diameter of the plants using a measuring tape. Shoots and roots were then carefully separated using a clean blade and weighed fresh in the field using a portable balance. Six of the twelve plants were dried in a forced-air oven and subsequently weighed to determine their dry biomass, which was then used to calculate the percentage of dry weight relative to the fresh weight. The LAI was assessed using an ACCUPAR ceptometer (LP 80, Meter Group, Pullman, WA, USA), with measurements taken above and below the canopy along a transect within each experimental plot. The remaining six plants were immediately placed in liquid nitrogen and transported to the laboratory under cold conditions. In the lab, samples were stored at –40 °C until analysis. Subsamples were later processed by lyophilization for phytohormone quantification, phenolic profiling, and mineral nutrient determination, while other portions of frozen material were used for oxidative stress and antioxidant assays.

### 4.3. Photosynthetic Pigments Concentration

The concentration of photosynthetic pigments was analyzed following the method of Wellburn [53], with slight modifications. A total of 0.1 g of the aerial part of frozen asparagus plants was macerated in 1 mL of methanol. The extract was then centrifuged for 5 min at 2200× *g*. Absorbance was measured at three different wavelengths: 653 nm, 666 nm, and 470 nm using a spectrophotometer (Infinite 200 Nanoquant, Tecan, Männedorf, Switzerland). Pigment concentrations were calculated as follows:

Chlorophyll a (Chl a) = 15.65 × A666 − 7.34 × A653

Chlorophyll b (Chl b) = 27.05 × A653 − 11.21 × A666

Carotenoids = (1000 × A470 − 2.86 × Chl a − 129.2 × Chl b)/221

### 4.4. Evaluation of Oxidative Metabolism and Antioxidant Tests

The concentration of MDA, was measured according to the Fu and Huang [54] method.

Superoxide dismutase enzyme (SOD) activity was determined using the method described by Yu et al. [55], based on the inhibition of the photochemical reduction of NBT and measured by spectrophotometry.

The H_2_O_2_ concentration was obtained according to the Junglee et al. [56] method.

The APX activity was determined by registering the change in absorbance at 290 nm over 3 min according to Yu et al. [55].

Total ascorbate (AsA) levels were determined following the method described by Law et al. [57], which is based on the reduction of ferric ions (Fe^3+^) by AsA and measured spectrophotometrically at 525 nm. Total glutathione (GSH) was quantified at 412 nm using the DTNB (5,5′-dithiobis-(2-nitrobenzoic acid)) method, as outlined by Noctor and Foyer [58], which detects thiol groups through the formation of a yellow-colored product.

The antioxidant capacity was further evaluated using two complementary assays. The Ferric Reducing Ability of Plasma (FRAP) assay was carried out following the protocol of Benzie and Strain [59], which assesses the reduction of Fe^3+^ to Fe^2+^ in the presence of antioxidants. The Trolox Equivalent Antioxidant Capacity (TEAC) assay was performed according to the method of Cai et al. [60], which measures the ability of antioxidants to quench the ABTS^+•^ radical cation.

All spectrophotometric measurements were conducted using an Infinite^®^ 200 NanoQuant microplate reader (Tecan, Switzerland).

### 4.5. Phytohormone Analysis

The quantification of phytohormones in the shoot tissue followed the methodology outlined by Albacete et al. [61], with minor adjustments. Approximately 30 mg of freeze-dried plant material was homogenized with 1 mL of a pre-chilled (−20 °C) methanol:water (80:20, *v*/*v*) extraction solution. The mixture was centrifuged at 20,000× *g* for 15 min at 4 °C. The pellet was re-extracted under the same conditions, and both supernatants were combined. The pooled extracts were purified using Sep-Pak Plus C18 cartridges (Waters, Milford, MA, USA). After drying, the residue was resuspended in 1 mL of methanol:water (20:80, *v*/*v*) in an ultrasonic bath. The resulting solution was passed through 0.22 μm nylon membrane filters (Millipore, Bedford, MA, USA) and 10 μL of the filtered extract was injected into a U-HPLC-MS system (ThermoFisher Scientific, Waltham, MA, USA) for phytohormone quantification.

### 4.6. Phenolic Compound Profiling

The profile of phenolic compounds in soil samples and freeze-dried root and shoot tissues was analyzed using both HPLC-DAD and HPLC-DAD-ESI-MS/MS techniques (Agilent Technologies, Waldbronn, 389 Germany). Plant tissues were extracted in methanol:water:formic acid (50:48:2) at a concentration of 100 mg/mL, whereas soil samples were treated with 80% methanol containing 2% triethylamine. Chromatographic separation was performed on a reversed-phase C18 Kinetex column (5 µm, 250 mm × 4.6 mm), employing a gradient elution with 1% formic acid in water and acetonitrile as the mobile phases. The flow rate was set to 0.8 mL/min and the injection volume was 20 µL. Spectral data were recorded between 200 and 600 nm, with chromatograms monitored at 330 nm. Phenolic acids (e.g., p-coumaric acid, caffeic acid and their glycosylated forms) and flavonoids (notably flavonol glycosides like apigenin diglucoside, kaempferol, quercetin, and isorhamnetin derivatives) were quantified using HPLC-DAD based on calibration curves prepared with pure standards. The identified compounds were categorized into phenolic acids and flavonoids. Chlorogenic acid (MW = 354) and rutin (MW = 628.5) served as reference compounds. Further structural elucidation was achieved via HPLC-DAD-ESI-MS/MS, employing an Agilent 1100 series system linked to an ion trap mass spectrometer equipped with an electrospray ionization (ESI) source. ESI conditions included a temperature of 350 °C, a capillary voltage of 4 kV, 60 psi nebulizer pressure, and a nitrogen flow rate of 11 L/min. Mass spectra were recorded in negative ionization mode over the *m*/*z* range of 100–1000. CID fragmentation was performed using helium. Compound identification was confirmed by comparing retention times, precursor ions, and fragmentation patterns with authentic standards and published data. MS/MS analysis was particularly important for distinguishing isomers or closely related compounds.

### 4.7. Determination of Mineral Elements

Elemental analysis was performed following the wet digestion protocol described by Wolf [62]. Lyophilized spear samples (0.1 g) were finely ground and digested using concentrated H_2_SO_4_ and H_2_O_2_ at 300 °C. Mineral content was measured via inductively coupled plasma optical emission spectrometry (ICP-OES; Perkin Elmer, Waltham, MA, USA) and expressed in mg or µg per gram of dry weight. For nitrogen determination, a separate digestion with H_2_SO_4_ and H_2_O_2_ was conducted using 0.1 g of dry tissue. The resulting solution was diluted with deionized water, and a 1 mL aliquot was added to a reaction mixture containing sodium silicate/sodium nitroprusside, sodium hydroxide, and sodium dichloroisocyanurate. The mixture was incubated at room temperature for 45 min, and nitrogen concentration was estimated by spectrophotometer (Infinite 200 Nanoquant, Tecan, Switzerland) following the procedure established by Krom [63].

### 4.8. Statistical Analysis

Statistical evaluation of treatment effects was conducted using one-way analysis of variance (ANOVA) with a 95% confidence threshold. Mean values and standard errors were calculated based on nine replicates per parameter. All statistical procedures were carried out using Statgraphics Centurion version 16.1.03.

## 5. Conclusions

In conclusion, among the soil disinfection treatments evaluated in this study, biofumigation with *B. carinata* pellets contributed to enhancing vegetative growth, photosynthetic pigment accumulation, antioxidant capacity, and hormonal balance in asparagus plants, with effects becoming more pronounced in the second year. This treatment promoted a healthier physiological status, likely through improving soil health and reducing biotic and abiotic stress perception, while chemical disinfection with dazomet, despite initially boosting certain physiological responses, was associated with increased oxidative stress and potential disruption of soil microbial communities. Overall, the results suggest that organic-based soil treatments, especially *B. carinata* biofumigation, offer a promising strategy for supporting asparagus vigor and resilience, although further studies are needed to assess their long-term effects in perennial cultivation systems.

## Figures and Tables

**Table 1:** Chlorophyll concentration, chlorophyll a/b ratio and carotenoid concentration in asparagus plants, subjected to the different treatments tested during two years of study (2020–2021).

		Total Chls mg g^−1^ FW	Chl a/b	Carotenoids mg g^−1^ FW
2020	T1	0.34 b	2.13 b	0.108 b
	T2	0.38 a	2.86 b	0.123 a
	T3	0.32 b	1.64 b	0.095 c
	T4	0.32 b	5.49 a	0.115 ab
	*p* value	*	***	***
2021	T1	0.20 c	1.50 b	0.059 c
	T2	0.33 a	2.15 a	0.104 a
	T3	0.25 b	2.13 a	0.081 b
	T4	0.28 b	2.15 a	0.093 b
	*p* value	***	***	***

Mean comparisons were performed using Fisher’s Least Significant Difference (LSD) test. Statistical significance is indicated as follows: * *p* < 0.05, *** *p* < 0.001, and NS for non-significant differences (*p* > 0.05). Different letters denote statistically significant differences between treatments. The treatments applied were: untreated control (T1), biosolarization with *Brassica* pellets (T2), biosolarization with poultry manure pellets (T3), and chemical soil disinfection using dazomet (T4).

**Table 2:** Concentration of phytohormones in the aerial part of asparagus, subjected to the different treatments tested during two years of study (2020–2021).

			Indole Acetic Acid	Trans-Zeatin	Zeatin Riboside	Isopentenyl Adenine	GA1	GA3	GA4	ACC	ABA	Jasmonic Acid	Salicylic Acid
Root	2020	T1	4.43	65.16	ND	28.73	ND	ND	1.37	189.48	2.20	6.45 ab	350.57
		T2	5.09	70.20	ND	18.33	ND	ND	0.82	260.95	2.08	5.03 b	375.38
		T3	4.78	83.68	ND	25.48	ND	ND	1.78	175.00	2.32	8.42 a	462.76
		T4	4.97	61.08	ND	22.91	ND	ND	0.46	280.85	2.73	6.66 ab	358.60
		*p* value	NS	NS	-	NS	-	-	NS	NS	NS	*	NS
	2021	T1	4.39	77.76	ND	21.19	ND	ND	0.51	237.18	1.11 b	6.47	287.86
		T2	4.68	89.02	ND	17.27	ND	ND	0.44	210.20	0.69 c	5.91	287.98
		T3	5.53	83.69	ND	23.95	ND	ND	0.61	209.74	1.14 b	6.09	271.5
		T4	4.86	91.97	ND	18.85	ND	ND	0.41	273.38	1.43 a	5.48	279.76
		*p* value	NS	NS	-	NS	-	-	NS	NS	***	NS	NS
Shoot	2020	T1	5.67 a	10.22	ND	3.81	ND	ND	0.02 b	28.00	40.87	31.45	102.63
		T2	4.04 c	13.09	ND	2.80	ND	ND	0.04 b	22.87	46.91	31.02	109.49
		T3	4.50 bc	11.10	ND	4.33	ND	ND	0.07 a	29.93	49.95	34.15	123.64
		T4	5.25 ab	12.31	ND	2.54	ND	ND	0.04 b	29.99	53.66	32.08	128.13
		*p* value	**	NS	-	NS	-	-	**	NS	NS	NS	NS
	2021	T1	8.22	9.85	ND	3.90	ND	ND	0.41	20.06	38.07	39.96 a	114.57 b
		T2	7.78	12.83	ND	4.09	ND	ND	0.29	24.28	40.92	28.67 b	125.73 a
		T3	9.71	9.69	ND	3.97	ND	ND	0.55	21.47	32.91	34.47 ab	115.02 b
		T4	9.83	11.48	ND	3.53	ND	ND	0.42	19.89	37.37	39.01 a	128.20 a
		*p* value	NS	NS	-	NS	-	-	NS	NS	NS	*	*

Mean comparisons were performed using Fisher’s Least Significant Difference (LSD) test. Statistical significance is indicated as follows: * *p* < 0.05, ** *p* < 0.01, *** *p* < 0.001, and NS for non-significant differences (*p* > 0.05). Different letters denote statistically significant differences between treatments. The treatments applied were: untreated control (T1), biosolarization with *Brassica* pellets (T2), biosolarization with poultry manure pellets (T3), and chemical soil disinfection using dazomet (T4). ND: non detected.

**Table 3:** Concentration of phenolic compounds in asparagus plants subjected to the different treatments tested during two years of study (2020–2021).

			4-CQA	Caffeic Acid Hexose	Caffeic Acid	Feruloil-QA	p-Coumaric Acid Derivative	Ferulic Acid Derivative	Apigenin Diglucoside (Vicenin2)	Q-Rutin-Hexose	Q-Rutin	Kaempferol Rutinoside	Quercetin Rutinoside Derivative	Isorhamnetin Rutinoside
Root	2020	T1	ND	0.74	3.40 ab	ND	ND	ND	ND	ND	ND	ND	ND	ND
		T2	ND	0.57	3.00 c	ND	ND	ND	ND	ND	ND	ND	ND	ND
		T3	ND	0.67	3.17 bc	ND	ND	ND	ND	ND	ND	ND	ND	ND
		T4	ND	0.57	3.58 a	ND	ND	ND	ND	ND	ND	ND	ND	ND
		*p* value	-	NS	**	-	-	-	-	-	-	-	-	-
	2021	T1	ND	0.88 a	3.52	ND	ND	ND	ND	ND	ND	ND	ND	ND
		T2	ND	0.69 ab	3.38	ND	ND	ND	ND	ND	ND	ND	ND	ND
		T3	ND	0.51 ab	3.55	ND	ND	ND	ND	ND	ND	ND	ND	ND
		T4	ND	0.39 b	3.96	ND	ND	ND	ND	ND	ND	ND	ND	ND
		*p* value	-	*	NS	-	-	-	-	-	-	-	-	-
Shoot	2020	T1	0	ND	ND	0.13 bc	0.43 b	0.12 b	1.93	0.27 b	9.70 b	0.56 a	0.20 a	0.16 b
		T2	0.05	ND	ND	0.08 c	0.41 b	0.10 b	1.56	0.56 a	8.95 c	0.44 b	0 b	0.10 b
		T3	0	ND	ND	0.22 a	0.47 b	0.12 b	1.62	0.16 b	9.48 bc	0.53 ab	0 b	0.36 a
		T4	0	ND	ND	0.16 ab	0.78 a	0.25 a	1.90	0.55 a	10.60 a	0.63 a	0.10 ab	0.43 a
		*p* value	*	-	-	**	***	**	NS	***	***	*	*	***
	2021	T1	0.19	ND	ND	ND	0.13	0.46 ab	1.04 a	ND	5.51 a	0.29	ND	ND
		T2	0.20	ND	ND	ND	0.09	0.31 b	0.78 b	ND	4.68 b	0.24	ND	ND
		T3	0.21	ND	ND	ND	0.16	0.61 a	0.85 b	ND	5.23 a	0.24	ND	ND
		T4	0.20	ND	ND	ND	0.09	0.33 b	0.71 b	ND	4.75 b	0.26	ND	ND
		*p* value	NS	-	-	-	NS	*	*	-	**	NS	-	-

Mean comparisons were performed using Fisher’s Least Significant Difference (LSD) test. Statistical significance is indicated as follows: * *p* < 0.05, ** *p* < 0.01, *** *p* < 0.001, and NS for non-significant differences (*p* > 0.05). Different letters denote statistically significant differences between treatments. The treatments applied were: untreated control (T1), biosolarization with *Brassica* pellets (T2), biosolarization with poultry manure pellets (T3), and chemical soil disinfection using dazomet (T4). ND: non detected.

**Table 4:** Concentration of mineral elements in asparagus plants subjected to the different treatments tested during two years of study (2020–2021).

			N	P	K	S	Ca	Mg	Fe	Mn	Zn	Cu	B
Root	2020	T1	99.12 a	1.30 a	17.37 b	3.14	4.12 b	1.85 c	1016.76	38.81	23.39	8.19	3.86
		T2	97.10 a	1.27 ab	20.90 a	3.39	6.10 a	2.38 ab	1611.04	44.57	23.84	8.35	4.19
		T3	73.81 b	1.11 bc	21.52 a	3.37	5.49 ab	1.70 c	1495.32	43.64	21.54	8.01	3.49
		T4	67.17 b	1.05 c	21.05 a	3.23	6.38 a	2.48 a	1486.81	45.91	22.99	7.43	4.28
		*p* value	***	**	*	NS	*	*	NS	NS	NS	NS	NS
	2021	T1	107.70 bc	1.33	20.08	4.23	7.01 b	1.80 b	219.61 b	56.75 b	27.71	8.16	27.25 ab
		T2	116.35 ab	1.27	19.16	3.68	7.64 a	2.00 ab	277.20 a	53.68 b	24.30	7.96	27.14 ab
		T3	128.14 a	1.30	20.53	4.02	7.28 b	2.21 a	195.70 b	55.84 b	23.02	7.68	24.10 b
		T4	92.89 c	1.34	20.41	4.34	8.69 a	2.25 a	305.6 a	73.92 a	23.18	8.37	32.52 a
		*p* value	**	NS	NS	NS	*	*	***	***	NS	NS	*
Shoot	2020	T1	97.81 a	1.15 a	12.26 a	2.97	5.29	1.69	2771.59	68.19	27.91 a	8.83	4.97
		T2	95.64 a	1.14 a	12.36 a	2.97	4.79	1.66	2545.04	64.22	26.32 a	8.13	4.53
		T3	75.20 b	1.08 ab	12.45 a	3.02	4.80	1.56	2482.70	62.51	25.67 ab	8.74	4.62
		T4	65.97 b	0.94 b	10.38 b	2.74	4.44	1.51	2481.12	63.21	22.36 b	7.98	3.77
		*p* value	**	*	***	NS	NS	NS	NS	NS	*	NS	NS
	2021	T1	109.26 a	1.80 a	14.87	4.13 a	5.37 a	0.79 b	192.59	58.81 a	31.79 a	8.25 a	36.33 a
		T2	115.89 a	1.86 a	14.70	3.40 b	3.50 b	1.04 a	151.57	36.16 c	29.37 a	7.36 b	19.67 c
		T3	127.51 a	1.76 ab	14.15	4.18 a	4.92 a	1.04 a	182.00	61.76 a	25.25 b	7.96 ab	30.52 ab
		T4	88.72 b	1.49 b	14.17	3.24 b	4.69 a	1.05 a	160.73	48.31 b	21.83 c	7.23 b	25.51 bc
		*p* value	**	*	NS	**	***	**	NS	***	***	*	***

N, P, K, S, Ca, Mg are expressed as mg g^−1^ DW, and Fe, Mn, Zn, Cu and B are expressed as µg g^−1^ DW. Mean comparisons were performed using Fisher’s Least Significant Difference (LSD) test. Statistical significance is indicated as follows: * *p* < 0.05, ** *p* < 0.01, *** *p* < 0.001, and NS for non-significant differences (*p* > 0.05). Different letters denote statistically significant differences between treatments. The treatments applied were: untreated control (T1), biosolarization with *Brassica* pellets (T2), biosolarization with poultry manure pellets (T3), and chemical soil disinfection using dazomet (T4).

## Data Availability

The datasets generated and analyzed during the current study are available from the corresponding author upon reasonable request.

## References

[1] Elmer W.H. (1996). Epidemiology and Management of the Diseases Causal to Asparagus Decline. Plant Dis..

[2] Knaflewski M. (1996). Genealogy of Asparagus Cultivars. Acta Hortic..

[3] Elmer W., Summerell B.A., Leslie J.F., Backhouse D. (2001). Fusarium Diseases of Asparagus. Paul E. Nelson Memorial Symposium.

[4] Baayen R.P., O’Donnell K., Bonants P.J.M., Cigelnik E., Kroon L.P.N.M., Roebroeck E.J.A., Waalwijk C. (2000). Gene Genealogies and AFLP Analyses in the *Fusarium Oxysporum* Complex Identify Monophyletic and Nonmonophyletic Formae Speciales Causing Wilt and Rot Disease. Phytopathology.

[5] Borrego-Benjumea A., Basallote-Ureba M.J., Melero-Vara J.M. Eficacia de Enmiendas Orgánicas, Temperatura de Suelo y Cultivares En El Control de La Podredumbre de Raíces y Cuello de Espárrago. Proceedings of the Resúmenes del XV Congreso de la Sociedad Española de Fitopatología, Vitoria-Gasteiz.

[6] Blok W.J., Bollen G.J. (1993). The Role of Autotoxins from Root Residues of the Previous Crop in the Replant Disease of Asparagus. Neth. J. Plant Pathol..

[7] Fischer U.A., Carle R., Kammerer D.R. (2011). Identification and Quantification of Phenolic Compounds from Pomegranate (*Punica granatum* L.) Peel, Mesocarp, Aril and Differently Produced Juices by HPLC-DAD–ESI/MSn. Food Chem..

[8] Schofield P.E. (1991). Asparagus Decline and Replant Problem in New Zealand. N. Z. J. Crop Hortic. Sci..

[9] Kato-Noguchi H., Nakamura K., Okuda N. (2018). Involvement of an Autotoxic Compound in Asparagus Decline. J. Plant Physiol..

[10] Kato-Noguchi H., Nakamura K., Ohno O., Suenaga K., Okuda N. (2017). Asparagus Decline: Autotoxicity and Autotoxic Compounds in Asparagus Rhizomes. J. Plant Physiol..

[11] Elmer W. (2018). Asparagus Decline and Replant Problem: A Look Back and a Look Forward at Strategies for Mitigating Losses. Acta Hortic..

[12] Corpas-Hervias C., Melero-Vara J.M., Molinero-Ruiz M.L., Zurera-Muñoz C., Basallote-Ureba M.J. (2006). Characterization of Isolates of *Fusarium* spp. Obtained from Asparagus in Spain. Plant Dis..

[13] Ito T., Ochiai T., Fukuda T., Ashizawa H., Kanno A., Kameya T., Sonoda T. (2008). Potential of Interspecific Hybrids in the Genus Asparagus. Acta Hortic..

[14] Kathe L., Krämer R., Budahn H., Pillen K., Rabenstein F., Nothnagel T. (2019). Development of a Bioassay to Assess Resistance to *Fusarium Oxysporum* (Schlecht.) in Asparagus (*Asparagus officinalis* L.). J. Phytopathol..

[15] Fravel D., Olivain C., Alabouvette C. (2003). *Fusarium Oxysporum* and Its Biocontrol. New Phytol..

[16] Bonanomi G., Antignani V., Pane C., Scala F. (2007). Suppression of Soilborne Fungal Diseases with Organic Amendments. J. Plant Pathol..

[17] dos Santos C.A., de Souza Abboud A.C., Carmo M.G.F.d. (2021). Biofumigation with Species of the Brassicaceae Family: A Review. Ciência Rural..

[18] Zhao R., Suo X., Meng X., Wang Y., Dai P., Hu T., Cao K., Wang S., Li B. (2024). Global Analysis of MicroRNA-like RNAs Reveals Differential Regulation of Pathogenicity and Development in Fusarium Oxysporum HS2 Causing Apple Replant Disease. J. Fungi.

[19] Wilson D.R., Sinton S.M., Butler R.C., Drost D.T., Paschold P.J., van Kruistum G., Poll J.T.K., Garcin C., Pertierra R., Vidal I. (2008). Carbohydrates and Yield Physiology of Asparagus—A Global Overview. Acta Hortic..

[20] Panth M., Hassler S.C., Baysal-Gurel F. (2020). Methods for Management of Soilborne Diseases in Crop Production. Agriculture.

[21] Hanschen F.S., Winkelmann T. (2020). Biofumigation for Fighting Replant Disease- A Review. Agronomy.

[22] Del Carmen Martínez-Ballesta M., Moreno D., Carvajal M. (2013). The Physiological Importance of Glucosinolates on Plant Response to Abiotic Stress in Brassica. Int. J. Mol. Sci..

[23] Nguyen V.P.T., Stewart J.D., Allais F., Ioannou I. (2022). Optimization of the Recovery of Secondary Metabolites from Defatted Brassica Carinata Meal and Its Effects on the Extractability and Functional Properties of Proteins. Foods.

[24] Yıldırım G.H., Ay E.B., Şengür Ş. (2024). Effects of Different Fertilizer Types on Pigment Content and Some Stress Molecules in Perennial Ryegrass (*Lolium perenne* L.). Legume Res.—An. Int. J..

[25] Muhammad I., Shalmani A., Ali M., Yang Q.-H., Ahmad H., Li F.B. (2021). Mechanisms Regulating the Dynamics of Photosynthesis Under Abiotic Stresses. Front. Plant Sci..

[26] Boutahiri S., Benrkia R., Tembeni B., Idowu O.E., Olatunji O.J. (2024). Effect of Biostimulants on the Chemical Profile of Food Crops under Normal and Abiotic Stress Conditions. Curr. Plant Biol..

[27] Gavelienė V., Mockevičiūtė R., Jankovska-Bortkevič E., Šveikauskas V., Zareyan M., Žalnierius T., Jankauskienė J., Jurkonienė S. (2025). Synergistic Effects of Microbial Biostimulants and Calcium in Alleviating Drought Stress in Oilseed Rape. Microorganisms.

[28] Kalaji H.M., Dąbrowski P., Cetner M.D., Samborska I.A., Łukasik I., Brestic M., Zivcak M. (2016). A Comparison between Different Chlorophyll Content Meters under Nutrients Deficiency Conditions. J. Plant Nutr..

[29] Bashri G., Prasad S.M. (2016). Exogenous IAA Differentially Affects Growth, Oxidative Stress and Antioxidants System in Cd Stressed Trigonella Foenum-Graecum L. Seedlings: Toxicity Alleviation by up-Regulation of Ascorbate-Glutathione Cycle. Ecotoxicol. Environ. Saf..

[30] Xiao M., Li Z., Zhu L., Wang J., Zhang B., Zheng F., Zhao B., Zhang H., Wang Y., Zhang Z. (2021). The Multiple Roles of Ascorbate in the Abiotic Stress Response of Plants: Antioxidant, Cofactor, and Regulator. Front. Plant Sci..

[31] Kumari V.V., Banerjee P., Verma V.C., Sukumaran S., Chandran M.A.S., Gopinath K.A., Venkatesh G., Yadav S.K., Singh V.K., Awasthi N.K. (2022). Plant Nutrition: An Effective Way to Alleviate Abiotic Stress in Agricultural Crops. Int. J. Mol. Sci..

[32] Hatamleh A.A., Danish M., Al-Dosary M.A., El-Zaidy M., Ali S. (2022). Physiological and Oxidative Stress Responses of *Solanum Lycopersicum* (L.) (Tomato) When Exposed to Different Chemical Pesticides. RSC Adv..

[33] Cuypers A., Vangronsveld J., Clijsters H. (2001). The Redox Status of Plant Cells (AsA and GSH) Is Sensitive to Zinc Imposed Oxidative Stress in Roots and Primary Leaves of Phaseolus Vulgaris. Plant Physiol. Biochem..

[34] Blázquez M.A., Nelson D.C., Weijers D. (2020). Evolution of Plant Hormone Response Pathways. Annu. Rev. Plant Biol..

[35] Rehman M., Saeed M.S., Fan X., Salam A., Munir R., Yasin M.U., Khan A.R., Muhammad S., Ali B., Ali I. (2023). The Multifaceted Role of Jasmonic Acid in Plant Stress Mitigation: An Overview. Plants.

[36] Li S., Liu S., Zhang Q., Cui M., Zhao M., Li N., Wang S., Wu R., Zhang L., Cao Y. (2022). The Interaction of ABA and ROS in Plant Growth and Stress Resistances. Front. Plant Sci..

[37] Cheynier V., Comte G., Davies K.M., Lattanzio V., Martens S. (2013). Plant Phenolics: Recent Advances on Their Biosynthesis, Genetics, and Ecophysiology. Plant Physiol. Biochem..

[38] Chowdhary V., Alooparampil S., Pandya R.V., Tank J.G. (2022). Physiological Function of Phenolic Compounds in Plant Defense System. Phenolic Compounds: Chemistry, Synthesis, Diversity, Non-Conventional Industrial, Pharmaceutical and Therapeutic Applications.

[39] Liu Q., Luo L., Zheng L. (2018). Lignins: Biosynthesis and Biological Functions in Plants. Int. J. Mol. Sci..

[40] Bailey K.L., Lazarovits G. (2003). Suppressing Soil-Borne Diseases with Residue Management and Organic Amendments. Soil. Tillage Res..

[41] Lattanzio V., Lattanzio V., Cardinali A. (2006). Role of Phenolics in the Resistance Mechanisms of Plants against Fungal Pathogens and Insects. Phytochem. Adv. Res..

[42] Agati G., Azzarello E., Pollastri S., Tattini M. (2012). Flavonoids as Antioxidants in Plants: Location and Functional Significance. Plant Sci..

[43] Friedman M. (2007). Overview of Antibacterial, Antitoxin, Antiviral, and Antifungal Activities of Tea Flavonoids and Teas. Mol. Nutr. Food Res..

[44] Sharma A., Shahzad B., Rehman A., Bhardwaj R., Landi M., Zheng B. (2019). Response of Phenylpropanoid Pathway and the Role of Polyphenols in Plants under Abiotic Stress. Molecules.

[45] Silva D.B., Turatti I.C.C., Gouveia D.R., Ernst M., Teixeira S.P., Lopes N.P. (2014). Mass Spectrometry of Flavonoid Vicenin-2, Based Sunlight Barriers in Lychnophora Species. Sci. Rep..

[46] Kim J.K., Park S.U. (2020). Recent Insights into the Biological Functions of Apigenin. EXCLI J..

[47] Marschner H. (2012). Mineral Nutrition of Higher Plants.

[48] Agegnehu G., Nelson P.N., Bird M.I. (2016). The Effects of Biochar, Compost and Their Mixture and Nitrogen Fertilizer on Yield and Nitrogen Use Efficiency of Barley Grown on a Nitisol in the Highlands of Ethiopia. Sci. Total Environ..

[49] Matthiessen J.N., Kirkegaard J.A. (2006). Biofumigation and Enhanced Biodegradation: Opportunity and Challenge in Soilborne Pest and Disease Management. CRC Crit. Rev. Plant Sci..

[50] Kirkegaard J.A., Sarwar M. (1998). Biofumigation Potential of Brassicas. Plant Soil..

[51] Lazzeri L., Leoni O., Manici L.M. (2004). Biocidal Plant Dried Pellets for Biofumigation. Ind. Crops Prod..

[52] Shennan C., Muramoto J., Lamers J., Mazzola M., Rosskopf E.N., Kokalis-Burelle N., Momma N., Butler D.M., Kobara Y. (2014). Anaerobic Soil Disinfestation for Soil Borne Disease Control in Strawberry and Vegetable Systems: Current Knowledge and Future Directions. Acta Hortic..

[53] Wellburn A.R. (1994). The Spectral Determination of Chlorophylls a and b, as Well as Total Carotenoids, Using Various Solvents with Spectrophotometers of Different Resolution. J. Plant Physiol..

[54] Fu J., Huang B. (2001). Involvement of Antioxidants and Lipid Peroxidation in the Adaptation of Two Cool-Season Grasses to Localized Drought Stress. Environ. Exp. Bot..

[55] Yu Q., Osborne L., Rengel Z. (1998). Micronutrient Deficiency Changes Activities of Superoxide Dismutase and Ascorbate Peroxidase in Tobacco Plants. J. Plant Nutr..

[56] Junglee S., Urban L., Sallanon H., Lopez-Lauri F. (2014). Optimized Assay for Hydrogen Peroxide Determination in Plant Tissue Using Potassium Iodide. Am. J. Anal. Chem..

[57] Law M.Y., Charles S.A., Halliwell B. (1983). Glutathione and Ascorbic Acid in Spinach (Spinacia Oleracea) Chloroplasts. The Effect of Hydrogen Peroxide and of Paraquat. Biochem. J..

[58] Noctor G., Foyer C.H. (1998). Simultaneous Measurement of Foliar Glutathione, γ-Glutamylcysteine, and Amino Acids by High-Performance Liquid Chromatography: Comparison with Two Other Assay Methods for Glutathione. Anal. Biochem..

[59] Benzie I.F.F., Strain J.J. (1996). The Ferric Reducing Ability of Plasma (FRAP) as a Aeasure of “Antioxidant Power”: The FRAP Assay. Anal. Biochem..

[60] Cai Y., Luo Q., Sun M., Corke H. (2004). Antioxidant Activity and Phenolic Compounds of 112 Traditional Chinese Medicinal Plants Associated with Anticancer. Life Sci..

[61] Albacete A., Ghanem M.E., Martinez-Andujar C., Acosta M., Sanchez-Bravo J., Martinez V., Lutts S., Dodd I.C., Perez-Alfocea F. (2008). Hormonal Changes in Relation to Biomass Partitioning and Shoot Growth Impairment in Salinized Tomato (*Solanum lycopersicum* L.) Plants. J. Exp. Bot..

[62] Wolf B. (1982). A Comprehensive System of Leaf Analyses and Its Use for Diagnosing Crop Nutrient Status. Commun. Soil. Sci. Plant Anal..

[63] Krom M.D. (1980). Spectrophotometric Determination of Ammonia: A Study of a Modified Berthelot Reaction Using Salicylate and Dichloroisocyanurate. Analyst.

